# GLP-1 Receptor Agonists at the Crossroads of Circadian Biology, Sleep, and Metabolic Disease

**DOI:** 10.3390/ijms27062853

**Published:** 2026-03-21

**Authors:** Ayush Gandhi, Ei Moe Phyu, Kwame Koom-Dadzie, Kodwo Bosomefi Dickson, Josiah Halm

**Affiliations:** Department of Hospital Medicine, The University of Texas MD Anderson Cancer Center, Houston, TX 77030, USA; ephyu@mdanderson.org (E.M.P.); kkoomdadzie@mdanderson.org (K.K.-D.); kbdickson@mdanderson.org (K.B.D.); jhalm@mdanderson.org (J.H.)

**Keywords:** GLP-1 receptor agonists, circadian rhythms, clock genes, chronotherapy, sleep disorder, metabolic disease, obstructive sleep apnea, type 2 diabetes, chronopharmacology, incretin hormones

## Abstract

Glucagon-like peptide-1 receptor agonists (GLP-1RAs) have transformed the management of type 2 diabetes and obesity, yet their actions extend beyond glycemic control and weight loss. This narrative review synthesizes current preclinical and clinical evidence examining the bidirectional relationship between glucagon-like peptide-1 (GLP-1) receptor agonists and circadian biology. A structured literature search was conducted in PubMed using combinations of the terms ‘GLP-1,’ ‘circadian,’ ‘chronobiology,’ ‘sleep,’ ‘obesity,’ and ‘type 2 diabetes’ through January 2026. Accumulating evidence indicates that GLP-1 physiology is closely coupled to circadian timing systems and sleep–wake regulation. In this narrative review, we synthesize emerging data that reframe GLP-1RAs as chronometabolic modulators, acting at the intersection of metabolism, circadian biology, and sleep. We review circadian control of GLP-1 secretion by intestinal L-cells, emphasizing the role of core clock genes and the vulnerability of incretin rhythms to circadian misalignment from shift work, nocturnal light exposure, and sleep loss. We then examine GLP-1 receptor signaling within central and peripheral clock networks, including feedback effects on hypothalamic and hepatic circadian regulation. Emerging data suggest that GLP-1 signaling is under circadian regulation and may, in turn, influence central and peripheral clock systems. Comparative discussion of semaglutide, liraglutide, and tirzepatide highlights agent-specific pharmacokinetics and emerging clinical data linking GLP-1RA therapy to sleep outcomes, particularly obstructive sleep apnea. Finally, we outline translational opportunities for chronotherapy and precision medicine, positioning GLP-1RAs as integrative tools for metabolic and sleep-related disease rather than purely weight-centric therapies. We propose that GLP-1 receptor agonists may function as chronometabolic modulators, with potential implications for personalized chronopharmacological strategies in metabolic disease.

## 1. Introduction

The incretin hormone GLP-1 is secreted by intestinal L-cells and plays a central role in metabolic regulation, most notably by augmenting glucose-stimulated insulin secretion and promoting satiety [1,2]. Beyond glycemia, GLP-1 receptors are widely distributed across the pancreas, central nervous system, and cardiovascular tissues, where they mediate diverse physiological effects, including cardioprotection, blood pressure reduction, and delayed gastric emptying [3]. On this basis, GLP-1 receptor agonists (GLP-1RAs), synthetic analogs of endogenous GLP-1, have become cornerstone therapies for type 2 diabetes and obesity [4].

Traditionally, these agents have been framed primarily as tools for lowering hemoglobin A1c and inducing weight loss. Increasingly, however, it has become clear that metabolism is not static across the day but is strongly shaped by circadian rhythms and sleep–wake organization [5,6]. Circadian clocks function as intrinsic 24 h timing systems, coordinated by the hypothalamic suprachiasmatic nucleus (SCN) and replicated across virtually all peripheral organs, orchestrating daily oscillations in physiology and behavior [6,7]. Sleep represents a major output of this system, and bidirectional relationships between sleep disruption and metabolic disease are now well established.

Modern lifestyles characterized by irregular nocturnal light exposure, shift work, late eating, and chronic sleep curtailment have exposed large segments of the population to persistent circadian disruption [8]. Such misalignment is increasingly recognized as a cardiometabolic risk factor, with shift workers experiencing approximately 20–35% higher risk of type 2 diabetes, cardiovascular disease, and certain cancers compared with day workers [5,6].

Against this background, it is notable that GLP-1 secretion itself is under circadian control, and that disruption of 24 h rhythmicity can impair incretin responses [1,2,9,10]. In humans, early metabolic studies demonstrated that the incretin response to identical meals varies by time of day, with significantly greater GLP-1 and GIP (Glucose-dependent Insulinotropic Polypeptide) release in the morning compared with the afternoon [1,9]. In healthy men, a single night of total sleep deprivation with nocturnal light exposure disrupted the normal timing of postprandial GLP-1 secretion, with peak response occurring later than under normal sleep conditions [1]. Over time, such temporal misalignment may contribute to impaired glucose tolerance and weight gain when feeding occurs at biologically suboptimal times.

Recent work has begun to bridge GLP-1 pharmacology with chronobiology. Preclinical studies have identified direct interactions between GLP-1 signaling and molecular clock machinery in tissues ranging from the gut to the brain [11,12,13]. Clinical observations further suggest that both the timing of GLP-1RA administration and underlying sleep patterns may influence therapeutic outcomes. Most notably, tirzepatide has recently received FDA approval for the treatment of moderate to severe obstructive sleep apnea (OSA) in adults with obesity, representing the first pharmacologic therapy approved specifically for OSA [14]. This decision was informed by the SURMOUNT-OSA trials, which demonstrated reductions in apnea–hypopnea index of approximately 20–24 events per hour compared with placebo [15,16], signaling a shift from purely symptomatic management toward disease-modifying intervention.

In this review, we integrate evidence from molecular biology, animal models, and human studies to frame GLP-1RAs as chronometabolic modulators. We first examine the circadian biology of GLP-1 secretion and its vulnerability to lifestyle-driven disruption. We then discuss how GLP-1RA therapy interfaces with circadian and sleep-regulatory systems, highlighting agent-specific differences where relevant. Despite advances in chronobiology and incretin pharmacotherapy, critical knowledge gaps remain. While preclinical studies suggest bidirectional interactions between GLP-1 signaling and molecular clock mechanisms, it is unknown whether these findings translate into clinically meaningful time-of-day differences in efficacy, tolerability, or cardiometabolic outcomes in humans. Furthermore, no prospective chronopharmacological trials have systematically evaluated whether GLP-1 receptor agonist administration timing modifies metabolic endpoints or sleep-related outcomes. Addressing these gaps is essential to determine whether circadian-informed prescribing strategies could enhance therapeutic precision.

Finally, we outline translational implications for chronotherapy and propose future research directions at the intersection of metabolism, circadian rhythms, and sleep.

## 2. Circadian and Sleep Regulation of Metabolism in Humans

Circadian rhythms and sleep exert powerful control over human metabolism at both systemic and cellular levels. Under physiological conditions, metabolic processes follow robust 24 h oscillations coordinated by the central clock in the SCN and reinforced by peripheral clocks in the liver, pancreas, skeletal muscle, and adipose tissue [6,7]. Insulin sensitivity and beta-cell responsiveness are generally highest in the biological morning, aligning metabolic capacity with typical feeding patterns, while glucose tolerance progressively declines later in the day [5,9,17].

Sleep, particularly consolidated nighttime sleep, represents a critical circadian output that feeds back on metabolic health. Adequate sleep supports insulin sensitivity and balanced regulation of appetite-related hormones, whereas sleep disruption promotes metabolic dysfunction [8]. Experimental studies have shown that even short-term sleep loss or nocturnal light exposure can impair glucose regulation. In one controlled study, a single night of sleeping with moderate room light increased insulin resistance the following morning and was accompanied by elevated nocturnal heart rate [1].

Circadian misalignment, defined as a mismatch between behavioral cycles and internal circadian timing, has become increasingly prevalent [18,19]. Shift workers provide a clear example, with epidemiologic studies consistently demonstrating a 1.17- to 1.25-fold increased risk of obesity and a 1.09- to 1.44- fold increase in type 2 diabetes among individuals engaged in long-term shift work [20,21,22]. Laboratory studies suggest that circadian misalignment is associated with increases in blood pressure, reductions in insulin sensitivity and secretion—sometimes approaching the prediabetic range, and elevations in postprandial lipid and glucose concentrations, although the magnitude and clinical durability of these effects vary across study designs [8,23,24].

Controlled human studies further demonstrate that postprandial glucose excursions are approximately 17% higher in the biological evening (8:00 p.m.) than in the biological morning (8:00 a.m.), independent of behavioral factors [17]. This circadian phase effect appears largely mediated by reduced pancreatic beta-cell function, with early-phase insulin secretion approximately 27% lower in the evening [25]. Circadian misalignment itself, such as a 12 h inversion of behavioral cycles, independently increases postprandial glucose by an additional 6%, likely through impaired insulin sensitivity [5,17,26,27,28].

Beyond glycemia, circadian and sleep disruption contribute to broader cardiometabolic risk. Chronic short sleep and irregular schedules are associated with elevated blood pressure and blunted diurnal blood pressure variation [29,30,31]. A meta-analysis of prospective studies found that short sleep (<6 h) was significantly associated with mortality, type 2 diabetes, hypertension, cardiovascular disease, coronary heart disease, and obesity [32]. Obstructive sleep apnea, characterized by sleep fragmentation and intermittent hypoxemia, is highly prevalent among individuals with obesity and type 2 diabetes [33,34]. Untreated OSA further exacerbates insulin resistance and hypertension, whereas effective treatment improves glycemic control in diabetic patients [35].

Taken together, these data underscore the importance of synchrony between the central clock, sleep–wake behavior, and metabolic processes in maintaining metabolic health. Disruption of this synchrony through shift work, poor sleep hygiene, or irregular eating patterns initiates a cascade of hormonal and metabolic disturbances that increase the risk of obesity, diabetes, and related conditions [36,37]. This framework provides critical context for understanding how therapies such as GLP-1RAs may interact with, and potentially restore, circadian–metabolic alignment.

## 3. Circadian Regulation of GLP-1 Secretion

### 3.1. Intrinsic Clock in L-Cells

Intestinal L-cells possess an intrinsic circadian clock that governs rhythmic GLP-1 secretion. Under physiological conditions, circulating GLP-1 exhibits clear diurnal variation. In rodents, secretion peaks near the onset of the feeding period and reaches a nadir during the fasting or rest phase [38]. Human studies mirror this pattern, with identical nutrient loads eliciting significantly greater GLP-1 and GIP release in the morning than in the afternoon [9]. In one study, early GLP-1 area under the curve (AUC_30_) was nearly twofold higher in the morning compared with the afternoon (300 ± 40 vs. 160 ± 30 pmol/L·30 min, *p* = 0.002) [9].

Mechanistic studies have confirmed that this rhythmicity depends on intact molecular clock machinery. In vivo, GLP-1 secretion parallels oscillations in the core clock gene Bmal1 (Arntl) within L-cells [39]. Targeted deletion or knockdown of Bmal1 in L-cells abolishes normal circadian variation in GLP-1 release, resulting in blunted secretion at the expected peak [13]. In Gcg-Arntl knockout mice, the rhythmic GLP-1 surge is lost, and peak GLP-1 levels are significantly reduced (*p* < 0.05), with downstream consequences including impaired timing of insulin secretion and disruption of intestinal homeostasis [12].

The circadian clock within intestinal L-cells governs time-of-day variation in GLP-1 secretion by directly regulating the exocytotic machinery required for hormone release. The core clock protein BMAL1 binds to promoter regions of genes encoding secretagogin (SCGN) and syntaxin-binding protein-1 (STXBP1)—SNARE-associated proteins that facilitate vesicle fusion with the cell membrane [40]. Knockdown of either *Scgn* or *Stxbp1* selectively impairs GLP-1 secretion at peak times while leaving baseline secretion unaffected (*p* < 0.01 and *p* < 0.05, respectively) [40]. In vivo, genetic deletion of *Scgn* using three independent knockout models abolishes the normal diurnal rhythm of GLP-1 secretion in response to oral glucose, with RNA-Seq revealing disrupted vesicle transport and synaptic protein interaction pathways [41]. These findings demonstrate that the molecular clock primes L-cells for optimal nutrient responsiveness at specific times of day through transcriptional control of secretory machinery.

Translational interpretation of these findings requires caution. Most mechanistic data derive from nocturnal rodent models, in which feeding, activity, and melatonin secretion are phase-inverted relative to diurnal humans. While the presence of circadian incretin regulation appears conserved, the temporal alignment of GLP-1 peaks and metabolic activity cannot be directly extrapolated from murine models to human physiology.

### 3.2. Metabolic Pathways Linking Clocks to Secretion

Beyond exocytotic control, the circadian clock also determines whether L-cells have sufficient metabolic capacity to secrete GLP-1. A central mechanism involves BMAL1-dependent regulation of nicotinamide adenine dinucleotide (NAD^+^) biosynthesis through nicotinamide phosphoribosyltransferase (NAMPT), the rate-limiting enzyme in the NAD^+^ salvage pathway [42,43]. In intact systems, Nampt expression and NAD^+^ availability oscillate with circadian timing, supporting time-of-day–dependent GLP-1 secretion. Disruption of this axis has clear functional consequences: suppression of Bmal1 or pharmacologic inhibition of NAMPT reduces NAD^+^ levels, impairs mitochondrial energy production, and blunts GLP-1 release [44]. Intestinal Nampt knockout mice develop reduced GLP-1 secretion, impaired early-phase insulin responses, and postprandial hyperglycemia, a phenotype reversible with NAD^+^ repletion using nicotinamide mononucleotide (NMN) [43]. Similar defects in intestinal NAD^+^ metabolism are observed in diet-induced obesity, paralleling impaired incretin secretion. Collectively, these findings support a clinically relevant BMAL1–NAMPT–NAD^+^ pathway linking circadian alignment to incretin competence, with implications for metabolic vulnerability in obesity, aging, and circadian disruption [42,45].

### 3.3. Impact of Nutritional Stress (Palmitate and High-Fat Diet)

The circadian GLP-1 axis appears particularly sensitive to dietary stress, especially exposure to saturated fatty acids common in Western diets. In rodent models, high-fat or high-sucrose feeding abolishes the normal diurnal rhythm of GLP-1 secretion, resulting in similar hormone levels at times when peak–trough differences would normally be expected [46]. Mechanistic studies suggest that palmitate, a dominant dietary saturated fatty acid, directly disrupts L-cell clock function by suppressing Bmal1 expression, reducing downstream Nampt-mediated NAD^+^ availability, and impairing mitochondrial ATP production required for hormone release (*p* < 0.05–0.001) [1,44]. Notably, palmitate fails to further suppress GLP-1 secretion in Bmal1-deficient L-cells, indicating that its primary target is the molecular clock rather than the secretory machinery itself [47]. Supporting this model, parallel work in hepatocytes shows that palmitate destabilizes BMAL1–CLOCK interactions via inhibition of SIRT1, a mechanism likely shared by intestinal L-cells [48]. With sustained exposure, palmitate also promotes ceramide-mediated apoptosis of GLP-1–producing cells, contributing to reduced L-cell mass in high-fat feeding states [49]. Collectively, these findings link dietary lipotoxicity to incretin dysfunction in obesity and suggest that obesogenic diets impair GLP-1 signaling not only through excess caloric load but by directly disrupting the circadian architecture that governs hormone release.

### 3.4. Mouse Models Supporting Circadian Control of GLP-1 Secretion

**Figure 1:**
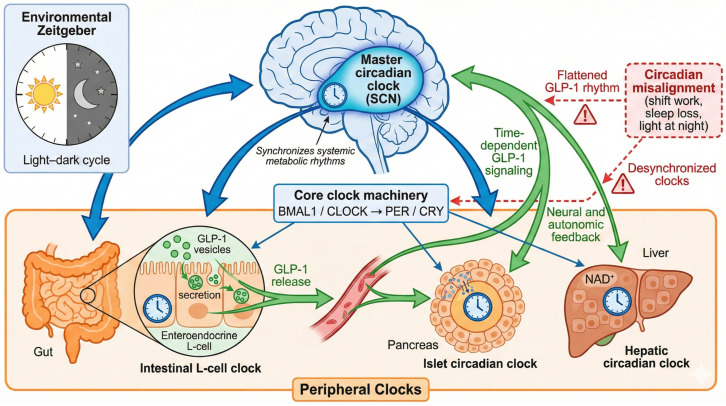
Circadian Regulation of GLP-1 Secretion and Systemic Action: A Conceptual Overview.

Preclinical knockout models provide important biological support for the clinical relevance of circadian regulation of GLP-1 secretion. In addition to L-cell–specific Bmal1 deletion, which directly establishes the requirement of BMAL1 for rhythmic GLP-1 release, mice lacking other core clock components (including Clock and Period genes) exhibit disrupted feeding behavior, impaired glucose homeostasis, and abnormal daily patterns of incretin secretion [13,19,50,51]. Although interpretation of germline Bmal1 knockout models is limited by global circadian disruption, the consistency of these findings across systems supports the concept that circadian control of GLP-1 is physiologically necessary [19,50,51]. L-cell clock disruption appears to extend beyond hormone secretion alone. Inducible L-cell Bmal1 knockout models demonstrate alterations in intestinal immune balance, gut microbial composition, and metabolite profiles, suggesting that rhythmic GLP-1 signaling may contribute to maintaining intestinal homeostasis [13]. From a clinical perspective, these data reinforce the idea that circadian misalignment and dietary disruption may impair GLP-1 biology at multiple levels, with downstream consequences for metabolic and gastrointestinal health. A conceptual overview of circadian regulation of GLP-1 secretion and its bidirectional interaction with central and peripheral clocks, as shown in Figure 1.

## 4. Circadian Misalignment and Incretin Dysfunction

### 4.1. Shift Work, Light at Night, and Sleep Deprivation

Circadian misalignment, whether from shift work, jet lag, or irregular light exposure, disrupts the normal 24 h pattern of GLP-1 secretion. Human studies confirm these findings. In healthy male volunteers, Gil-Lozano and colleagues demonstrated that identical meals elicited significantly greater GLP-1 responses when consumed at 11:00 a.m. compared with 11:00 p.m., establishing a clear diurnal pattern in human L-cell activity [1]. Critically, sleep deprivation combined with nocturnal light exposure induced profound derangements in GLP-1 and insulin responses, abrogating the normal time-of-day variation in incretin secretion [1]. These alterations were not observed in sleep-deprived participants maintained under dark conditions, indicating a direct effect of light rather than sleep loss alone on glucoregulatory pathways [3]. Phase-delayed circadian misalignment in controlled laboratory settings similarly decreases GLP-1 concentrations (*p* = 0.02) while increasing glucose levels, demonstrating that even modest shifts in circadian timing impair incretin-glucose coordination [52]. Metabolic regulation is influenced not only by the timing of light exposure but also by the 24 h amplitude of light. Robust daytime natural light strengthens circadian entrainment and metabolic oscillation amplitude, whereas diminished daytime exposure combined with artificial evening light can flatten circadian rhythms [53]. This reduced light amplitude, common in modern indoor lifestyles, may independently impair glucose and lipid homeostasis and could potentially blunt endogenous incretin rhythmicity [54,55].

These findings carry direct clinical relevance. Shift workers experience chronic exposure to light at night, suppressed melatonin rhythms, and mistimed feeding all factors that disrupt the circadian GLP-1 axis [6,56]. The resulting “scrambled” incretin profile, characterized by blunted morning GLP-1 responses and delayed postprandial peaks, mirrors the metabolic phenotype observed in epidemiological studies linking shift work to a 20–35% increased risk of type 2 diabetes. Long-term sleep deprivation in primate models similarly induces insulin resistance associated with impaired GLP-1 signaling, effects that can be partially attenuated by probiotic supplementation targeting the gut microbiome [57]. Collectively, these data suggest that circadian disruption represents a modifiable pathway through which modern lifestyle factors such as irregular light exposure, curtailed sleep, and mistimed eating contribute to incretin dysfunction and metabolic disease risk.

### 4.2. Microbiota and Rhythmic GLP-1 Secretion

We now recognize the gut microbiome as essential for circadian GLP-1 secretion. Microbiome depletion in germ-free or antibiotic-treated mice completely abolishes the postprandial GLP-1 response, an effect restored by fecal microbiota transplantation [2,58]. Specific taxa, including *Ruminococcaceae* and *Lachnospiraceae* oscillate diurnally alongside intestinal clock gene expression, and this coordination is disrupted in type 2 diabetic models with impaired GLP-1 sensitivity [7]. Mechanistically, gut bacteria regulate GLP-1 through multiple pathways: *Akkermansia muciniphila* secretes a protein (P9) that induces GLP-1 release via ICAM-2 interaction in an IL-6–dependent manner, while microbial bile acid metabolism, particularly ω-muricholic acid and hyocholic acid, stimulates L-cells through TGR5 signaling [59,60]. Short-chain fatty acids (SCFA) from bacterial fermentation bind free fatty acid receptors 2 and 3 on L-cells to promote GLP-1 secretion, though the nuclear receptor FXR can inhibit this pathway by reducing FFAR2 expression [61,62]. In humans, circulating short-chain fatty acid (SCFA) concentrations are typically in the low micromolar range (e.g., acetate ≈ 20–80 µmol/L, propionate ≈ 1–13 µmol/L, butyrate ≈ 0.5–5 µmol/L in fasting plasma) have been positively associated with fasting GLP-1 levels in observational cohorts, suggesting that microbial metabolites reaching the systemic circulation may be more directly linked to incretin function than fecal concentrations alone [63]. These findings suggest that a eubiotic microbiome supports robust circadian GLP-1 secretion, whereas dysbiosis, as occurs in obesity and type 2 diabetes, may dampen or desynchronize incretin rhythms, opening opportunities for prebiotic, probiotic, or synbiotic co-treatment strategies to enhance GLP-1-based therapies [11].

### 4.3. Other Factors Affecting GLP-1 Rhythms

Beyond light, sleep, and the microbiome, we recognize several additional modulators of circadian GLP-1 secretion with clinical relevance. Meal timing exerts powerful effects: time-restricted feeding promotes GLP-1 secretion and regulates appetite through enhanced Lactobacillus colonization and tryptophan-derived indole-3-lactic acid production, which increases enteroendocrine cell differentiation [64]. Conversely, erratic meal patterns and obesogenic diets render GLP-1 secretion arrhythmic, eliminating the normal peak–trough differences essential for coordinated metabolic responses [2]. Glucocorticoids represent another critical modulator: dexamethasone suppresses GLP-1 secretion both in vitro and in vivo through glucocorticoid receptor activation in L-cells, reducing proglucagon expression at the transcriptional level via a receptor dimerization-dependent mechanism [65]. This bidirectional relationship is notable because GLP-1 itself activates the hypothalamic–pituitary–adrenal (HPA) axis, increasing circulating cortisol in humans and corticosterone in rodents. These effects are observed with both endogenous GLP-1 and therapeutic GLP-1 receptor agonists [65]. Chronic stress and elevated glucocorticoids may therefore blur GLP-1 rhythmicity through both direct L-cell suppression and central feedback mechanisms, potentially contributing to the metabolic dysregulation observed in stress-related disorders.

In summary, we recognize that circadian misalignment arising from abnormal light exposure, sleep loss, shift work, or microbiome disruption impairs the coordinated timing of GLP-1 secretion required for metabolic homeostasis. These findings suggest that clinical strategies and trial designs should move beyond dose-centric approaches and begin to account for when GLP-1 signaling is engaged, including attention to dosing time, sleep–wake patterns, and circadian context, as part of precision metabolic therapy.

## 5. GLP-1 Receptor Signaling in Central and Peripheral Clock Systems

### 5.1. Central GLP-1 Pathways and the Master Clock

Beyond its peripheral actions, we recognize GLP-1 as an important neuropeptide linking feeding behavior to central circadian control. GLP-1–producing preproglucagon (PPG) neurons in the caudal nucleus tractus solitarius (NTS) project extensively to hypothalamic regions involved in both appetite and circadian regulation, including the paraventricular nucleus (PVN), arcuate nucleus (ARC), and dorsomedial hypothalamus (DMH) [66,67,68]. The DMH serves as a key relay between feeding-related signals and the master clock in the suprachiasmatic nucleus (SCN). Experimental disruption of GLP-1 receptor signaling in the DMH leads to loss of normal diurnal feeding patterns, hyperphagia, weight gain, and blunted circadian metabolic rhythms [69]. GLP-1 signaling in this pathway appears necessary for conveying meal timing information to the SCN. Together, these data position central GLP-1 signaling as a critical mechanism by which feeding schedules help entrain circadian rhythms of behavior and metabolism [67].

### 5.2. Peripheral Clock Feedback via GLP-1 Receptor Agonists

In addition to being regulated by circadian clocks, we find that GLP-1 signaling can itself influence clock timing in peripheral tissues. Preclinical studies show that GLP-1 receptor agonists modulate peripheral clock gene expression through mechanisms distinct from refeeding; for example, exendin-4 alters hepatic *Per1* mRNA expression and modifies feeding-induced clock gene changes in the liver and adipose tissue [70]. These effects are strongly time-dependent: exenatide administered at the beginning of the active period (ZT 12) counteracts mistimed feeding–induced phase shifts in the hepatic clock, whereas dosing at the rest period onset (ZT 0) exacerbates circadian disruption [71]. Importantly, these clock-modulating effects are largely lost in mice with central nervous system–specific GLP-1 receptor knockdown, indicating that peripheral clock resetting occurs indirectly through brain-mediated pathways rather than direct hepatic action [71]. Beyond the liver, GLP-1 mimetics such as liraglutide and exenatide induce high-amplitude rhythmic PER2 expression in pancreatic β-cells via adenylate cyclase–dependent signaling, highlighting cell-specific clock modulation [72]. From a translational perspective, these findings suggest that GLP-1RA dosing time may be clinically relevant, particularly in patients with irregular eating patterns or circadian disruption, providing a biological rationale for GLP-1–based chronotherapy.

In summary, we recognize that GLP-1 functions not only as a gut-derived incretin but also as a neuromodulator linking metabolic signals to central circadian control. Through GLP-1 receptors in the dorsomedial hypothalamus and related hypothalamic nuclei, incretin signaling helps align feeding behavior with the master clock, as disruption of this pathway abolishes normal diurnal feeding patterns and blunts meal-induced activation of the suprachiasmatic nucleus [69,73]. Beyond the brain, timed activation of GLP-1 receptor pathways can modify peripheral clock timing: exenatide administered during the active phase attenuates mistimed feeding–induced phase shifts in the liver, whereas dosing during the rest phase exacerbates circadian disruption via centrally mediated mechanisms [70,71]. In pancreatic islets, GLP-1 mimetics such as liraglutide and exenatide also induce robust, rhythmic PER2 expression in β-cells, demonstrating cell-specific clock synchronization [72]. Taken together, these central and peripheral actions position GLP-1 receptor agonists as potential tools for addressing circadian disruption. We next compare semaglutide, liraglutide, and tirzepatide with respect to how they may differentially engage these chronometabolic pathways, including effects on sleep.

## 6. Agent-Specific Effects of GLP-1 Receptor Agonists

### 6.1. Pharmacokinetics, Dosing Frequency, and Circadian Exposure

GLP-1 receptor agonists differ meaningfully in pharmacokinetics, central engagement, and downstream effects on feeding behavior, with potential implications for chronometabolic regulation [74]. Liraglutide, with a half-life of 11–15 h, produces daily peaks and troughs and is more amenable to time-of-day tailoring, whereas semaglutide (half-life ~165–168 h) and tirzepatide (half-life ~117 h) provide near-continuous receptor engagement across the 24 h cycle with once-weekly dosing [75,76]. This sustained exposure may blunt late-day hunger and reduce nocturnal eating, reinforcing circadian-aligned feeding patterns; both agents reduce food cravings and binge eating, with semaglutide lowering binge-eating prevalence and liraglutide improving eating-disorder risk scores versus placebo [77].

Evidence on receptor desensitization is reassuring but nuanced. Although chronic GLP-1RA exposure induces homologous receptor desensitization in vitro, GLP-1R–dependent glucose homeostasis appears preserved in vivo [78]. A human pilot study likewise showed no tolerance to liraglutide’s glucose-lowering effect over 21 days [79]. Mechanistically, GLP-1 receptors are functionally Gs-selective and recycle between membrane microdomains rather than undergoing classical β-arrestin–mediated desensitization, which may explain the durability of long-acting agents [74,80]. Nonetheless, tolerance develops for some effects such as gastric motility, and one murine study reported loss of glucose-lowering efficacy after 18 days, underscoring potential species- or dose-dependent differences [77].

The metabolic effects of GLP-1 receptor agonists extend beyond weight loss. In a randomized controlled trial, Mashayekhi et al. showed that liraglutide improved insulin sensitivity by HOMA-IR, HOMA2, and the Matsuda index—within two weeks, before meaningful weight reduction occurred [81]. These effects were GLP-1 receptor–dependent, as they were reversed by exendin [9,39]. In contrast, diet-induced weight loss of similar magnitude improved HOMA-based indices but not the Matsuda index or fasting glucose, suggesting that GLP-1 receptor agonists engage pathways beyond simple caloric restriction [81,82].

Clinically, this early, weight-independent insulin sensitization suggests that GLP-1 therapy may begin restoring metabolic alignment before significant weight loss, potentially strengthening the reciprocal relationship between glucose control, sleep, and circadian stability.

### 6.2. Central Nervous System Penetration

All three agents, namely liraglutide, semaglutide, and tirzepatide acts on GLP-1 receptors accessible from the periphery, particularly within brainstem regions such as the area postrema and nucleus tractus solitarius, which lie outside a fully intact blood–brain barrier and are central to appetite regulation and nausea [83,84]. Experimental data indicate that the nucleus tractus solitarius is a key site of action, as selective GLP-1 receptor knockdown in this region attenuates the anorectic and weight-reducing effects of liraglutide [83,84].

Liraglutide also gains access to hypothalamic feeding centers via tanycyte-mediated transport rather than classical blood–brain barrier penetration. Disruption of this pathway abolishes hypothalamic neuronal activation and the drug’s effects on food intake and body weight [85]. Within the arcuate nucleus, liraglutide activates anorexigenic POMC/CART neurons and suppresses orexigenic NPY/AgRP signaling, providing a neural basis for its weight-loss efficacy [86]. GLP-1 receptors are present in the human hypothalamus and brainstem, and liraglutide alters central responses to highly palatable food cues in individuals with diabetes [87].

Semaglutide appears to engage similar central pathways, with preferential hypothalamic accumulation, although available data suggest that acylated GLP-1 analogs act primarily through circumventricular access and specialized transport rather than direct blood–brain barrier penetration [74,88]. Its longer half-life likely enables more sustained central receptor engagement.

In contrast, tirzepatide’s central nervous system penetration is less well characterized, and much of its additional efficacy is thought to derive from peripheral mechanisms and dual GLP-1/GIP receptor signaling [89]. The extent to which tirzepatide directly crosses the intact blood–brain barrier remains uncertain, and any central effects may reflect limited penetration, signaling at circumventricular organs, or indirect modulation through peripheral vagal pathways. While GIP receptors are expressed in the brain, whether tirzepatide engages them centrally at physiological concentrations and with what relevance to circadian or behavioral regulation remains uncertain [89]. Definitive pharmacokinetic studies quantifying CNS exposure in humans are lacking.

### 6.3. Appetite Circadian Patterns

GLP-1 receptor agonists produce sustained reductions in appetite and caloric intake through central satiety pathways and improved control of eating behavior [78,90,91]. In clinical trials, semaglutide 2.4 mg significantly improved craving control across multiple food domains, with reductions in craving scores correlating with weight loss over long-term follow-up [92]. Oral semaglutide 50 mg reduced ad libitum energy intake by nearly 40% at 20 weeks, alongside lower hunger and food cravings [93]. Tirzepatide similarly reduced energy intake early in treatment and was associated with marked decreases in appetite, food cravings, and overeating tendencies [94]. In head-to-head comparisons, tirzepatide and semaglutide both reduced appetite versus placebo, with comparable effects on energy intake despite greater weight loss with tirzepatide [95].

With respect to eating timing, direct evidence that GLP-1RAs specifically reduce late-night or nocturnal eating remains limited. Semaglutide has been shown to markedly reduce emotional eating and craving prevalence, suggesting broad improvement in maladaptive eating behaviors rather than targeted effects on meal timing [96]. From a circadian nutrition perspective, the sustained appetite suppression of long-acting agents may indirectly favor earlier caloric intake during periods of higher insulin sensitivity, although this remains hypothesis-generating and requires prospective, time-stamped dietary studies. Liraglutide’s shorter half-life (~13 h) raises the theoretical possibility of time-of-day tailoring, but current clinical guidance supports consistent daily dosing rather than circadian-directed administration [75,97].

### 6.4. Sleep Architecture Effects

From a clinical standpoint, the most consistent sleep-related benefit of GLP-1 receptor agonists is improvement in obstructive sleep apnea (OSA). However, it is essential to recognize that this benefit is largely, if not entirely, mediated through weight loss rather than direct pharmacologic action on sleep physiology. Weight reduction decreases pharyngeal fat deposition, reduces mechanical load on the upper airway, and improves respiratory mechanics, which are effects that would be expected with any intervention producing comparable weight loss. Whether GLP-1RAs exert any weight-independent effects on OSA severity remains unestablished. Given the high burden of OSA in obesity and type 2 diabetes, this represents a clinically meaningful downstream effect of incretin-based therapy. In the SCALE Sleep Apnea trial, liraglutide 3.0 mg produced a significantly greater reduction in apnea–hypopnea index (AHI) than placebo over 32 weeks, with improvements closely linked to weight reduction [98]. Tirzepatide appears to confer the largest OSA benefit to date: in the SURMOUNT-OSA trials, tirzepatide reduced AHI by approximately 20–24 events per hour versus placebo, both in patients using positive airway pressure therapy and those not receiving PAP [14]. These improvements were accompanied by reduced hypoxic burden, lower inflammatory markers, and better patient-reported sleep outcomes. A recent meta-analysis of randomized trials further supports a class effect, demonstrating a mean AHI reduction of 11.6 events per hour with incretin-based therapies compared with usual care [75].

Beyond OSA, direct effects of GLP-1 receptor agonists on human sleep architecture remain poorly defined. While preclinical studies suggest potential sleep-promoting effects, human data are limited, and reported improvements in subjective sleep quality likely reflect weight loss and OSA improvement rather than primary neuromodulatory actions [99]. Sleep-related adverse effects, including insomnia or fragmented sleep, are uncommon and usually related to gastrointestinal symptoms. At present, available clinical evidence does not support intentional use of GLP-1 receptor agonists to directly modify sleep architecture or circadian sleep timing independent of their metabolic effects. Table 1 summarizes agent-specific pharmacokinetic, central, circadian, and sleep-related considerations for commonly used GLP-1 receptor agonists

## 7. Chronotherapy and Precision Medicine with GLP-1RA Therapy

Given the close interplay between circadian biology and GLP-1 signaling, chronotherapy may represent a promising extension of incretin-based treatment. Preclinical data suggest that the timing of GLP-1 receptor agonist administration may influence metabolic alignment: in mice subjected to circadian misalignment through light-phase feeding, exenatide administered at the onset of the active period (ZT 12) counteracted the phase-shifting effect on the hepatic clock, whereas dosing at the rest-phase onset (ZT 0) exacerbated circadian disruption [71]. These clock-modulating effects were largely abolished in mice with central nervous system–specific GLP-1 receptor knockdown, indicating that the timing effects are mediated through brain GLP-1 signaling rather than direct peripheral action [71]. In humans, morning administration may better align with endogenous insulin sensitivity, GLP-1 secretion, and feeding-related signaling, while minimizing overlap with sleep. Current prescribing guidance for liraglutide recommends administration ‘at any time of day, although timing should be consistent,’ reflecting practical adherence considerations rather than circadian optimization. This flexibility may inadvertently support personalized timing approaches, as patients can select administration times that align with their individual schedules and circadian patterns, though evidence-based guidance for such individualization is currently lacking. No randomized controlled trials have directly compared morning versus evening dosing of GLP-1 receptor agonists to determine whether administration timing influences glycemic control, weight loss, or circadian metabolic outcomes.

**Figure 2:**
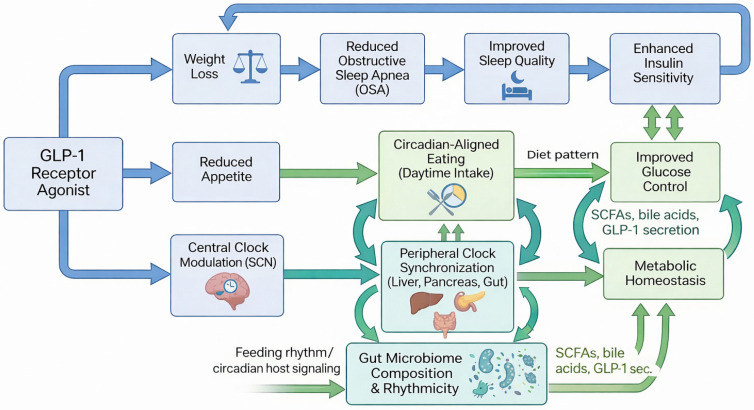
Clinical Integration Model of GLP-1 Receptor Agonists in Circadian–Metabolic Health.

Beyond dosing time, precision approaches may integrate GLP-1 receptor agonist therapy with meal timing, chronotype, and behavioral circadian interventions. Long-acting agents such as semaglutide and tirzepatide, with half-lives exceeding 100 h, provide near-continuous receptor engagement that may reduce late-night appetite and indirectly promote circadian-aligned eating patterns [106,107]. In contrast, liraglutide’s shorter half-life (~13 h) theoretically permits greater temporal tailoring, though this remains unexplored in clinical trials. Combining GLP-1 receptor agonists with strategies such as time-restricted feeding, morning light exposure, or structured exercise may further reinforce metabolic rhythms, although direct evidence for synergistic effects is lacking. Future trials incorporating continuous glucose monitoring, wearable sleep and activity metrics, and chronotype stratification will be critical to define which patients benefit most from chronotherapy-guided use. Ultimately, we envision that aligning GLP-1 receptor agonist treatment with an individual’s biological and behavioral rhythms offers a pragmatic path toward more precise and durable metabolic care. The integrated clinical pathways through which GLP-1 receptor agonists influence metabolic and circadian health are summarized in Figure 2.

## 8. Translational and Clinical Implications

The convergence of circadian biology, sleep physiology, and GLP-1 receptor agonist therapy carries important implications for both research and clinical practice. Reframing these agents beyond a purely weight-centric lens allows us to approach metabolic disease more holistically. Clinically, improvements in glycemic control and weight loss with GLP-1 receptor agonists are often accompanied by better sleep and circadian alignment. Reduced obstructive sleep apnea events, less nocturnal reflux, and improved daytime energy can create a reinforcing cycle in which better sleep enhances insulin sensitivity and appetite regulation, further supporting metabolic control [82,108]. Given that weight loss is a cornerstone of OSA management and that CPAP (Continuous Positive Airway Pressure) adherence remains suboptimal in many patients, GLP-1 receptor agonists may increasingly serve as adjunctive or alternative therapy for obesity-related sleep-disordered breathing [109,110]. Notably, tirzepatide is now FDA-approved for the treatment of moderate-to-severe OSA in adults with obesity, representing the first pharmacotherapy specifically indicated for this condition [110]. This emerging overlap highlights the potential for closer collaboration between endocrinology and sleep medicine.

A circadian perspective also encourages a broader “24 h” approach to care. Rather than focusing solely on fasting labs or HbA1c, we may benefit from assessing diurnal glucose patterns, nocturnal blood pressure behavior, and sleep–wake regularity when initiating GLP-1 receptor agonist therapy. Continuous glucose monitoring reveals that GLP-1 receptor agonists reduce glucose variability and time spent in hyperglycemic ranges across the 24 h cycle, with the combination of basal insulin and GLP-1 receptor agonist producing the lowest glucose variability and hypoglycemia among common insulin regimens [111]. Wearable sleep and activity trackers can help ensure that metabolic improvements are accompanied by healthier circadian patterns rather than inadvertent disruption. Looking forward, integrating chronotherapy principles with GLP-1 receptor agonist use alongside meal timing, light exposure, and sleep optimization may enhance the durability of benefit and inform future drug development aimed at reinforcing both metabolic and circadian health.

### 8.1. Clinical Implications

In current clinical practice, we can already apply several actionable strategies when initiating GLP-1 receptor agonist therapy. First, we should assess patient schedules. Asking about work hours, meal timing, and sleep habits helps contextualize dosing recommendations, particularly for patients with nontraditional schedules such as shift work, even though formal evidence for circadian-directed dosing remains limited.

Second, we should monitor tolerability in relation to the time of day. Common gastrointestinal adverse effects such as nausea, vomiting, diarrhea, and constipation typically occur during dose escalation. When symptoms arise, it is reasonable to assess whether they cluster around dosing time and consider timing adjustments alongside standard mitigation strategies such as slower up-titration, smaller meals, avoidance of high-fat foods, and adequate hydration [112,113]. However, the temporal dynamics of these adverse effects remain under-characterized. Whether these symptoms exhibit circadian clustering in relation to gastric emptying rhythms or postprandial hormonal peaks is unknown. Understanding the chronobiology of adverse effects may provide an opportunity to optimize dosing time to improve adherence without compromising efficacy.

Third, we should emphasize routine. Consistent daily or weekly dosing, coupled with regular meal timing and sleep schedules, aligns with current guidance and may synergize with metabolic effects. Anchoring medication administration to stable habits such as a morning routine or regular breakfast can also support adherence and circadian regularity.

Fourth, we should actively leverage weight loss to improve sleep. As patients lose weight, we should reassess symptoms of obstructive sleep apnea and overall sleep quality. Many patients experience reduced snoring and improved daytime alertness, and some may be able to down-titrate or discontinue PAP (Positive Airway Pressure) therapy under supervision. Persistent fatigue despite weight loss should prompt evaluation for residual or alternative sleep disorders.

Finally, incorporating simple sleep and circadian assessments into metabolic care may improve outcomes. Brief tools such as STOP-BANG or the Pittsburgh Sleep Quality Index can identify patients who warrant further evaluation. Emerging data also suggest that adequate sleep duration may amplify glycemic benefits of GLP-1 receptor signaling, with gene–environment studies showing interactions between GLP-1 receptor variants and sleep duration on HbA1c [53]. Addressing sleep regularity alongside GLP-1RA therapy may therefore enhance durability of response and long-term adherence.

### 8.2. Limitations

This review has several limitations. This review is narrative in structure and does not employ a formal systematic search strategy, predefined inclusion/exclusion criteria, or quantitative meta-analysis. As such, it may be subject to selection bias, and the magnitude of effect sizes across heterogeneous studies cannot be formally compared. The intent of this work is hypothesis-generating and integrative rather than quantitatively definitive. Additionally, much of the mechanistic evidence derives from preclinical models, and translational applicability to humans remains uncertain. Clinical data directly evaluating time-of-day administration of GLP-1 receptor agonists are limited, and most available studies were not designed as chronopharmacological trials. Also to note, associations between GLP-1 signaling, sleep disorders, and microbiome alterations are largely observational, precluding causal inference. Existing clinical trials have employed fixed dosing schedules, leaving the chronotherapeutic hypothesis largely speculative. No randomized controlled trials have directly compared different times of day for GLP-1RA administration or stratified outcomes by patient chronotype or circadian phase markers. Furthermore, much of the mechanistic foundation derives from nocturnal animal models, limiting direct chronological translation to human clinical practice. Finally, heterogeneity in study populations, endpoints, and dosing regimens limits cross-study comparability.

### 8.3. Future Directions

Finally, we see several clinically relevant directions for future work at the intersection of circadian biology and GLP-1 receptor agonist therapy. A key unanswered question is whether GLP-1 receptor agonists meaningfully influence circadian clock function in humans, as suggested by preclinical studies showing time-dependent effects on peripheral clock gene expression. Determining whether such effects occur in human tissues and whether they translate into better metabolic control would directly inform clinical use.

From a practical standpoint, combining GLP-1 receptor agonists with circadian-targeted interventions warrants careful study. Strategies such as melatonin supplementation or timed light exposure can improve circadian alignment and sleep quality in selected populations, including shift workers, but their metabolic effects are variable and context dependent. Whether pairing these approaches with GLP-1RA therapy improves glycemic or weight outcomes remains unknown and should be tested in well-designed clinical trials. Critically, future trials should move beyond simple morning-versus-evening comparisons to test truly personalized chronotherapy, for example, matching GLP-1RA administration to individual circadian phenotypes assessed via chronotype questionnaires, actigraphy, or circadian biomarkers such as dim-light melatonin onset.

We also need trials that reflect real-world circadian diversity. Current outcome studies have not examined whether responses to GLP-1 receptor agonists differ by chronotype, shift-work status, or circadian phase markers. Incorporating tools already familiar to clinicians—such as actigraphy and time-stamped continuous glucose monitoring could help identify patients who benefit most from timing-informed therapy.

## 9. Conclusions

GLP-1 receptor agonists have become core therapies for diabetes and obesity, and it is now evident that their effects extend beyond glucose and weight into circadian biology and sleep regulation. As we have discussed, GLP-1 is not an isolated metabolic hormone but part of the body’s 24 h timing system. Circadian clocks in the gut regulate GLP-1 secretion, and disruption of these rhythms through irregular sleep, abnormal light exposure, or mistimed eating can impair metabolic control. In turn, GLP-1 signaling in the brain and periphery can feed back on circadian systems, providing potential leverage to mitigate circadian misalignment.

Across agents, including liraglutide, semaglutide, and tirzepatide, clinically relevant interactions with circadian physiology are apparent, whether through effects on feeding behavior, peripheral clock regulation, or improvement in obstructive sleep apnea, largely mediated by weight loss. These insights encourage a more time-aware approach to metabolic care. For agents that influence appetite, insulin secretion, and inflammation, timing may determine whether therapy simply lowers glucose or more broadly supports metabolic alignment. In practice, it may be reasonable to incorporate circadian considerations by assessing patients’ sleep and meal patterns and encouraging regularity alongside GLP-1 receptor agonist therapy. Ultimately, integrating circadian biology into GLP-1 receptor agonist therapy moves us toward a more integrative and personalized metabolic medicine, one that treats disease in synchrony with the patient’s internal clock.

## Figures and Tables

**Table 1:** Chronometabolic and Sleep-Relevant Differences Among Common GLP-1 Receptor Agonists.

Agent	Dosing & PK	Central Engagement	Mechanistic Circadian Effects	Circadian Clinical Implications	Sleep/OSA Outcomes
Liraglutide	Daily; t½ ~13 h [100]	Acts at AP/NTS; limited hypothalamic access [101]	Intermittent receptor exposure may better preserve physiologic rhythmicity; preclinical and conceptual support for time-of-day responsiveness [10]	Allows potential time-of-day tailoring, although this has not been tested in prospective chronopharmacology trials	Improves OSA severity primarily in association with weight loss (~12 AHI/h); whether any benefit is weight-independent remains uncertain
Semaglutide	Weekly; t½ ~160 h [102]	Sustained brainstem/hypothalamic GLP-1R activation [103]	Near-continuous receptor signaling may blunt physiologic circadian variation in GLP-1 tone; clinical relevance remains uncertain [46]	Fixed weekly exposure may reduce opportunities for meaningful dosing-time optimization; no human evidence yet shows time-of-day differences in efficacy or tolerability	OSA benefit is expected mainly through weight reduction; no direct evidence currently demonstrates sleep-stage effects or weight-independent OSA benefit
Tirzepatide	Weekly; t½ ~120 h [104]	GLP-1R engagement; central GIPR effects remain incompletely defined [105]	Continuous dual-agonist signaling may influence circadian metabolic regulation indirectly, but agent-specific circadian mechanisms remain poorly defined	Chronotherapeutic implications remain speculative; no prospective trials have evaluated whether timing of administration alters metabolic or sleep outcomes	Greatest OSA improvement reported (~20–24 AHI/h), likely driven predominantly by weight loss; possible weight-independent effects remain unproven

Abbreviations: t½, elimination half-life; NTS, nucleus tractus solitarius; AP, area postrema; OSA, obstructive sleep apnea; AHI, apnea–hypopnea index; PK, pharmacokinetics.

## Data Availability

No new data were created or analyzed in this study. Data sharing is not applicable to this article.

## Abbreviations

The following abbreviations are used in this manuscript:

AHI	Apnea–Hypopnea Index
AgRP	Agouti-Related Peptide
ARC	Arcuate Nucleus
AP	Area Postrema
BMI	Body Mass Index
BMAL1	Brain and Muscle ARNT-Like 1
BBB	Blood–Brain Barrier
CART	Cocaine- and Amphetamine-Regulated Transcript
CGM	Continuous Glucose Monitoring
CI	Confidence Interval
CLOCK	Circadian Locomotor Output Cycles Kaput
CPAP	Continuous Positive Airway Pressure
CNS	Central Nervous System
Dbp	D-box Binding PAR bZIP Transcription Factor
DMH	Dorsomedial Hypothalamus
GIP	Glucose-Dependent Insulinotropic Polypeptide
GIPR	Glucose-Dependent Insulinotropic Polypeptide Receptor
GLP-1	Glucagon-Like Peptide-1
GLP-1R	Glucagon-Like Peptide-1 Receptor
GLP-1RA	Glucagon-Like Peptide-1 Receptor Agonist
HbA1c	Hemoglobin A1c
HOMA-IR	Homeostatic Model Assessment of Insulin Resistance
HOMA2	Updated Homeostatic Model Assessment
L-cells	Enteroendocrine L-cells
NAMPT	Nicotinamide Phosphoribosyltransferase
NAD^+^	Nicotinamide Adenine Dinucleotide
NREM	Non-Rapid Eye Movement
NPY	Neuropeptide Y
NTS	Nucleus Tractus Solitarius
OSA	Obstructive Sleep Apnea
PAP	Positive Airway Pressure
PER	Period Protein
POMC	Proopiomelanocortin
RCT	Randomized Controlled Trial
SCN	Suprachiasmatic Nucleus
STOP-BANG	Snoring, Tiredness, Observed Apnea, Blood Pressure, BMI, Age, Neck Circumference, Gender
TRF	Time-Restricted Feeding
ZT	Zeitgeber Time

## References

[1] Gil-Lozano M., Hunter P.M., Behan L.-A., Gladanac B., Casper R.F., Brubaker P.L. (2016). Short-Term Sleep Deprivation with Nocturnal Light Exposure Alters Time-Dependent Glucagon-like Peptide-1 and Insulin Secretion in Male Volunteers. Am. J. Physiol. Endocrinol. Metab..

[2] Martchenko S.E., Martchenko A., Cox B.J., Naismith K., Waller A., Gurges P., Sweeney M.E., Philpott D.J., Brubaker P.L. (2020). Circadian GLP-1 Secretion in Mice Is Dependent on the Intestinal Microbiome for Maintenance of Diurnal Metabolic Homeostasis. Diabetes.

[3] Pyke C., Heller R.S., Kirk R.K., Ørskov C., Reedtz-Runge S., Kaastrup P., Hvelplund A., Bardram L., Calatayud D., Knudsen L.B. (2014). GLP-1 Receptor Localization in Monkey and Human Tissue: Novel Distribution Revealed with Extensively Validated Monoclonal Antibody. Endocrinology.

[4] Kalyani R.R. (2021). Glucose-Lowering Drugs to Reduce Cardiovascular Risk in Type 2 Diabetes. N. Engl. J. Med..

[5] Morris C.J., Purvis T.E., Mistretta J., Scheer F.A.J.L. (2016). Effects of the Internal Circadian System and Circadian Misalignment on Glucose Tolerance in Chronic Shift Workers. J. Clin. Endocrinol. Metab..

[6] Meyer N., Harvey A.G., Lockley S.W., Dijk D.-J. (2022). Circadian Rhythms and Disorders of the Timing of Sleep. Lancet.

[7] Grasset E., Puel A., Charpentier J., Klopp P., Christensen J.E., Lelouvier B., Servant F., Blasco-Baque V., Tercé F., Burcelin R. (2022). Gut Microbiota Dysbiosis of Type 2 Diabetic Mice Impairs the Intestinal Daily Rhythms of GLP-1 Sensitivity. Acta Diabetol..

[8] Kervezee L., Kosmadopoulos A., Boivin D.B. (2020). Metabolic and Cardiovascular Consequences of Shift Work: The Role of Circadian Disruption and Sleep Disturbances. Eur. J. Neurosci..

[9] Lindgren O., Mari A., Deacon C.F., Carr R.D., Winzell M.S., Vikman J., Ahrén B. (2009). Differential Islet and Incretin Hormone Responses in Morning versus Afternoon after Standardized Meal in Healthy Men. J. Clin. Endocrinol. Metab..

[10] Gil-Lozano M., Mingomataj E.L., Wu W.K., Ridout S.A., Brubaker P.L. (2014). Circadian Secretion of the Intestinal Hormone GLP-1 by the Rodent L Cell. Diabetes.

[11] Zeng Y., Wu Y., Zhang Q., Xiao X. (2024). Crosstalk between Glucagon-like Peptide 1 and Gut Microbiota in Metabolic Diseases. mBio.

[12] Biancolin A.D., Martchenko A., Mitova E., Gurges P., Michalchyshyn E., Chalmers J.A., Doria A., Mychaleckyj J.C., Adriaenssens A.E., Reimann F. (2020). The Core Clock Gene, Bmal1, and Its Downstream Target, the SNARE Regulatory Protein Secretagogin, Are Necessary for Circadian Secretion of Glucagon-like Peptide-1. Mol. Metab..

[13] Martchenko S.E., Martchenko A., Biancolin A.D., Waller A., Brubaker P.L. (2021). L-Cell Arntl Is Required for Rhythmic Glucagon-like Peptide-1 Secretion and Maintenance of Intestinal Homeostasis. Mol. Metab..

[14] Malhotra A., Grunstein R.R., Fietze I., Weaver T.E., Redline S., Azarbarzin A., Sands S.A., Schwab R.J., Dunn J.P., Chakladar S. (2024). Tirzepatide for the Treatment of Obstructive Sleep Apnea and Obesity. N. Engl. J. Med..

[15] Garvey W.T., Frias J.P., Jastreboff A.M., le Roux C.W., Sattar N., Aizenberg D., Mao H., Zhang S., Ahmad N.N., Bunck M.C. (2023). Tirzepatide Once Weekly for the Treatment of Obesity in People with Type 2 Diabetes (SURMOUNT-2): A Double-Blind, Randomised, Multicentre, Placebo-Controlled, Phase 3 Trial. Lancet.

[16] Malhotra A., Bednarik J., Chakladar S., Dunn J.P., Weaver T., Grunstein R., Fietze I., Redline S., Azarbarzin A., Sands S.A. (2024). Tirzepatide for the Treatment of Obstructive Sleep Apnea: Rationale, Design, and Sample Baseline Characteristics of the SURMOUNT-OSA Phase 3 Trial. Contemp. Clin. Trials.

[17] Morris C.J., Yang J.N., Garcia J.I., Myers S., Bozzi I., Wang W., Buxton O.M., Shea S.A., Scheer F.A.J.L. (2015). Endogenous Circadian System and Circadian Misalignment Impact Glucose Tolerance via Separate Mechanisms in Humans. Proc. Natl. Acad. Sci. USA.

[18] Finger A.-M., Kramer A. (2021). Mammalian Circadian Systems: Organization and Modern Life Challenges. Acta Physiol..

[19] Allada R., Bass J. (2021). Circadian Mechanisms in Medicine. N. Engl. J. Med..

[20] Kecklund G., Axelsson J. (2016). Health Consequences of Shift Work and Insufficient Sleep. BMJ.

[21] Gao Y., Gan T., Jiang L., Yu L., Tang D., Wang Y., Li X., Ding G. (2020). Association between Shift Work and Risk of Type 2 Diabetes Mellitus: A Systematic Review and Dose-Response Meta-Analysis of Observational Studies. Chronobiol. Int..

[22] Liu Q., Shi J., Duan P., Liu B., Li T., Wang C., Li H., Yang T., Gan Y., Wang X. (2018). Is Shift Work Associated with a Higher Risk of Overweight or Obesity? A Systematic Review of Observational Studies with Meta-Analysis. Int. J. Epidemiol..

[23] Scheer F.A.J.L., Hilton M.F., Mantzoros C.S., Shea S.A. (2009). Adverse Metabolic and Cardiovascular Consequences of Circadian Misalignment. Proc. Natl. Acad. Sci. USA.

[24] Wefers J., van Moorsel D., Hansen J., Connell N.J., Havekes B., Hoeks J., van Marken Lichtenbelt W.D., Duez H., Phielix E., Kalsbeek A. (2018). Circadian Misalignment Induces Fatty Acid Metabolism Gene Profiles and Compromises Insulin Sensitivity in Human Skeletal Muscle. Proc. Natl. Acad. Sci. USA.

[25] Qian J., Dalla Man C., Morris C.J., Cobelli C., Scheer F.A.J.L. (2018). Differential Effects of the Circadian System and Circadian Misalignment on Insulin Sensitivity and Insulin Secretion in Humans. Diabetes Obes. Metab..

[26] Calvo-Malvar M., Lado-Baleato Ó., Ríos A.C., Fernández C.P., Benítez-Calvo A., Fernandez-Merino C., Sánchez-Castro J., Wagner R., Matabuena M., Gude F. (2025). Age, Sex, BMI, Meal Timing, and Glycemic Response to Meal Glycemic Load. JAMA Netw. Open.

[27] Hatamoto Y., Tanoue Y., Yoshimura E., Matsumoto M., Hayashi T., Ogata H., Tanaka S., Tanaka H., Higaki Y. (2023). Delayed Eating Schedule Raises Mean Glucose Levels in Young Adult Males: A Randomized Controlled Cross-Over Trial. J. Nutr..

[28] Leung G.K.W., Huggins C.E., Bonham M.P. (2019). Effect of Meal Timing on Postprandial Glucose Responses to a Low Glycemic Index Meal: A Crossover Trial in Healthy Volunteers. Clin. Nutr..

[29] Haghayegh S., Strohmaier S., Hamaya R., Eliassen A.H., Willett W.C., Rimm E.B., Schernhammer E.S. (2023). Sleeping Difficulties, Sleep Duration, and Risk of Hypertension in Women. Hypertension.

[30] Qi J., Yang M., Zhang S., He C., Bao X., He B., Lin Y., Chu J., Chen K. (2025). The Association Between Sleep Duration and the Risk of Hypertension: A Systematic Review and Meta-Analysis of Cohort Studies. J. Gen. Intern. Med..

[31] Hosseini K., Soleimani H., Tavakoli K., Maghsoudi M., Heydari N., Farahvash Y., Etemadi A., Najafi K., Askari M.K., Gupta R. (2024). Association between Sleep Duration and Hypertension Incidence: Systematic Review and Meta-Analysis of Cohort Studies. PLoS ONE.

[32] Javaheri S., Javaheri S., Somers V.K., Gozal D., Mokhlesi B., Mehra R., McNicholas W.T., Zee P.C., Campos-Rodriguez F., Martinez-Garcia M.A. (2024). Interactions of Obstructive Sleep Apnea with the Pathophysiology of Cardiovascular Disease, Part 1: JACC State-of-the-Art Review. J. Am. Coll. Cardiol..

[33] Subramanian A., Adderley N.J., Tracy A., Taverner T., Hanif W., Toulis K.A., Thomas G.N., Tahrani A.A., Nirantharakumar K. (2019). Risk of Incident Obstructive Sleep Apnea Among Patients with Type 2 Diabetes. Diabetes Care.

[34] Drager L.F., Togeiro S.M., Polotsky V.Y., Lorenzi-Filho G. (2013). Obstructive Sleep Apnea: A Cardiometabolic Risk in Obesity and the Metabolic Syndrome. J. Am. Coll. Cardiol..

[35] Herth J., Sievi N.A., Schmidt F., Kohler M. (2023). Effects of Continuous Positive Airway Pressure Therapy on Glucose Metabolism in Patients with Obstructive Sleep Apnoea and Type 2 Diabetes: A Systematic Review and Meta-Analysis. Eur. Respir. Rev..

[36] Ansu Baidoo V., Knutson K.L. (2023). Associations between Circadian Disruption and Cardiometabolic Disease Risk: A Review. Obesity.

[37] Schrader L.A., Ronnekleiv-Kelly S.M., Hogenesch J.B., Bradfield C.A., Malecki K.M. (2024). Circadian Disruption, Clock Genes, and Metabolic Health. J. Clin. Investig..

[38] Segers A., Depoortere I. (2021). Circadian Clocks in the Digestive System. Nat. Rev. Gastroenterol. Hepatol..

[39] Liu C., Liu Y., Xin Y., Wang Y. (2022). Circadian Secretion Rhythm of GLP-1 and Its Influencing Factors. Front. Endocrinol..

[40] Campbell J.R., Martchenko A., Sweeney M.E., Maalouf M.F., Psichas A., Gribble F.M., Reimann F., Brubaker P.L. (2020). Essential Role of Syntaxin-Binding Protein-1 in the Regulation of Glucagon-Like Peptide-1 Secretion. Endocrinology.

[41] Biancolin A.D., Srikrishnaraj A., Jeong H., Martchenko A., Brubaker P.L. (2022). The Cytoskeletal Transport Protein, Secretagogin, Is Essential for Diurnal Glucagon-like Peptide-1 Secretion in Mice. Endocrinology.

[42] Nagahisa T., Kosugi S., Yamaguchi S. (2023). Interactions between Intestinal Homeostasis and NAD+ Biology in Regulating Incretin Production and Postprandial Glucose Metabolism. Nutrients.

[43] Nagahisa T., Yamaguchi S., Kosugi S., Homma K., Miyashita K., Irie J., Yoshino J., Itoh H. (2022). Intestinal Epithelial NAD+ Biosynthesis Regulates GLP-1 Production and Postprandial Glucose Metabolism in Mice. Endocrinology.

[44] Martchenko A., Oh R.H., Wheeler S.E., Gurges P., Chalmers J.A., Brubaker P.L. (2018). Suppression of Circadian Secretion of Glucagon-like Peptide-1 by the Saturated Fatty Acid, Palmitate. Acta Physiol..

[45] Stein L.R., Imai S. (2012). The Dynamic Regulation of NAD Metabolism in Mitochondria. Trends Endocrinol. Metab..

[46] Martchenko A., Brubaker P.L. (2021). Effects of Obesogenic Feeding and Free Fatty Acids on Circadian Secretion of Metabolic Hormones: Implications for the Development of Type 2 Diabetes. Cells.

[47] Mukherji A., Kobiita A., Ye T., Chambon P. (2013). Homeostasis in Intestinal Epithelium Is Orchestrated by the Circadian Clock and Microbiota Cues Transduced by TLRs. Cell.

[48] Tong X., Zhang D., Arthurs B., Li P., Durudogan L., Gupta N., Yin L. (2015). Palmitate Inhibits SIRT1-Dependent BMAL1/CLOCK Interaction and Disrupts Circadian Gene Oscillations in Hepatocytes. PLoS ONE.

[49] Thombare K., Ntika S., Wang X., Krizhanovskii C. (2017). Long Chain Saturated and Unsaturated Fatty Acids Exert Opposing Effects on Viability and Function of GLP-1-Producing Cells: Mechanisms of Lipotoxicity. PLoS ONE.

[50] Marcheva B., Ramsey K.M., Buhr E.D., Kobayashi Y., Su H., Ko C.H., Ivanova G., Omura C., Mo S., Vitaterna M.H. (2010). Disruption of the Clock Components CLOCK and BMAL1 Leads to Hypoinsulinaemia and Diabetes. Nature.

[51] Perelis M., Marcheva B., Ramsey K.M., Schipma M.J., Hutchison A.L., Taguchi A., Peek C.B., Hong H., Huang W., Omura C. (2015). Pancreatic β Cell Enhancers Regulate Rhythmic Transcription of Genes Controlling Insulin Secretion. Science.

[52] Gonnissen H.K.J., Rutters F., Mazuy C., Martens E.A.P., Adam T.C., Westerterp-Plantenga M.S. (2012). Effect of a Phase Advance and Phase Delay of the 24-h Cycle on Energy Metabolism, Appetite, and Related Hormones. Am. J. Clin. Nutr..

[53] Wei J., Wu H., Zheng Y., Wang N., Benedict C., Chen W., Tan X. (2024). Adequate Sleep Duration Accentuates the Effect of Glucagon-like Peptide-1 Receptor Variant on HbA1c: A Gene-Environment Interaction Study. Diabetes Res. Clin. Pract..

[54] Biller A.M., Balakrishnan P., Spitschan M. (2024). Behavioural determinants of physiologically-relevant light exposure. Commun. Psychol..

[55] Fleury G., Masís-Vargas A., Kalsbeek A. (2020). Metabolic implications of exposure to light at night: Lessons from animal and human studies. Obesity.

[56] Meléndez-Fernández O.H., Liu J.A., Nelson R.J. (2023). Circadian Rhythms Disrupted by Light at Night and Mistimed Food Intake Alter Hormonal Rhythms and Metabolism. Int. J. Mol. Sci..

[57] Zhao Y., Shu Y., Zhao N., Zhou Z., Jia X., Jian C., Jin S. (2022). Insulin Resistance Induced by Long-Term Sleep Deprivation in Rhesus Macaques Can Be Attenuated by Bifidobacterium. Am. J. Physiol. Endocrinol. Metab..

[58] Wang Q., Lin H., Shen C., Zhang M., Wang X., Yuan M., Yuan M., Jia S., Cao Z., Wu C. (2023). Gut Microbiota Regulates Postprandial GLP-1 Response via Ileal Bile Acid-TGR5 Signaling. Gut Microbes.

[59] Cani P.D., Knauf C. (2021). A Newly Identified Protein from Akkermansia Muciniphila Stimulates GLP-1 Secretion. Cell Metab..

[60] Yoon H.S., Cho C.H., Yun M.S., Jang S.J., You H.J., Kim J.-H., Han D., Cha K.H., Moon S.H., Lee K. (2021). Akkermansia Muciniphila Secretes a Glucagon-like Peptide-1-Inducing Protein That Improves Glucose Homeostasis and Ameliorates Metabolic Disease in Mice. Nat. Microbiol..

[61] Tolhurst G., Heffron H., Lam Y.S., Parker H.E., Habib A.M., Diakogiannaki E., Cameron J., Grosse J., Reimann F., Gribble F.M. (2012). Short-Chain Fatty Acids Stimulate Glucagon-like Peptide-1 Secretion via the G-Protein-Coupled Receptor FFAR2. Diabetes.

[62] Ducastel S., Touche V., Trabelsi M.-S., Boulinguiez A., Butruille L., Nawrot M., Peschard S., Chávez-Talavera O., Dorchies E., Vallez E. (2020). The Nuclear Receptor FXR Inhibits Glucagon-Like Peptide-1 Secretion in Response to Microbiota-Derived Short-Chain Fatty Acids. Sci. Rep..

[63] Müller M., Hernández M.A.G., Goossens G.H., Reijnders D., Holst J.J., Jocken J.W.E., van Eijk H., Canfora E.E., Blaak E.E. (2019). Circulating but Not Faecal Short-Chain Fatty Acids Are Related to Insulin Sensitivity, Lipolysis and GLP-1 Concentrations in Humans. Sci. Rep..

[64] Li Q., Tan D., Xiong S., Yu K., Su Y., Zhu W. (2025). Time-Restricted Feeding Promotes Glucagon-like Peptide-1 Secretion and Regulates Appetite via Tryptophan Metabolism of Gut Lactobacillus in Pigs. Gut Microbes.

[65] Sato T., Hayashi H., Hiratsuka M., Hirasawa N. (2015). Glucocorticoids Decrease the Production of Glucagon-like Peptide-1 at the Transcriptional Level in Intestinal L-Cells. Mol. Cell. Endocrinol..

[66] Diz-Chaves Y., Herrera-Pérez S., González-Matías L.C., Lamas J.A., Mallo F. (2020). Glucagon-Like Peptide-1 (GLP-1) in the Integration of Neural and Endocrine Responses to Stress. Nutrients.

[67] Hwang E., Portillo B., Williams K.W. (2025). Glucagon-Like Peptide 1 (GLP-1) Action on Hypothalamic Feeding Circuits. Endocrinology.

[68] Gu G., Roland B., Tomaselli K., Dolman C.S., Lowe C., Heilig J.S. (2013). Glucagon-like Peptide-1 in the Rat Brain: Distribution of Expression and Functional Implication. J. Comp. Neurol..

[69] Maejima Y., Yokota S., Shimizu M., Horita S., Kobayashi D., Hazama A., Shimomura K. (2021). The Deletion of Glucagon-like Peptide-1 Receptors Expressing Neurons in the Dorsomedial Hypothalamic Nucleus Disrupts the Diurnal Feeding Pattern and Induces Hyperphagia and Obesity. Nutr. Metab..

[70] Ando H., Ushijima K., Fujimura A. (2013). Indirect Effects of Glucagon-like Peptide-1 Receptor Agonist Exendin-4 on the Peripheral Circadian Clocks in Mice. PLoS ONE.

[71] Xu P., Morishige J.-I., Jing Z., Nagata N., Shi Y., Iba T., Daikoku T., Ono M., Maida Y., Fujiwara T. (2024). Exenatide Administration Time-Dependently Affects the Hepatic Circadian Clock through Glucagon-like Peptide-1 Receptors in the Central Nervous System. Biochem. Pharmacol..

[72] Petrenko V., Dibner C. (2018). Cell-Specific Resetting of Mouse Islet Cellular Clocks by Glucagon, Glucagon-like Peptide 1 and Somatostatin. Acta Physiol..

[73] Huang Z., Liu L., Zhang J., Conde K., Phansalkar J., Li Z., Yao L., Xu Z., Wang W., Zhou J. (2022). Glucose-Sensing Glucagon-like Peptide-1 Receptor Neurons in the Dorsomedial Hypothalamus Regulate Glucose Metabolism. Sci. Adv..

[74] Gandhi A., Parhizgar A. (2025). GLP-1 Receptor Agonists in Alzheimer’s and Parkinson’s Disease: Endocrine Pathways, Clinical Evidence, and Future Directions. Front. Endocrinol..

[75] Gudzune K.A., Kushner R.F. (2024). Medications for Obesity: A Review. JAMA.

[76] Rubino D.M., Greenway F.L., Khalid U., O’Neil P.M., Rosenstock J., Sørrig R., Wadden T.A., Wizert A., Garvey W.T., STEP 8 Investigators (2022). Effect of Weekly Subcutaneous Semaglutide vs. Daily Liraglutide on Body Weight in Adults with Overweight or Obesity Without Diabetes: The STEP 8 Randomized Clinical Trial. JAMA.

[77] Jebeile H., Danielsen Y.S., Sumithran P., Lorien S., Jardine I.R., Baur L.A., Lister N.B. (2025). GLP-1 Receptor Agonist Medications for Obesity and Type 2 Diabetes Treatment: A Rapid Review of Changes in Eating Behaviors and Eating Disorder Risk. Obes. Rev..

[78] Alfaris N., Waldrop S., Johnson V., Boaventura B., Kendrick K., Stanford F.C. (2024). GLP-1 Single, Dual, and Triple Receptor Agonists for Treating Type 2 Diabetes and Obesity: A Narrative Review. eClinicalMedicine.

[79] Wilcox T., De Block C., Schwartzbard A.Z., Newman J.D. (2020). Diabetic Agents, from Metformin to SGLT2 Inhibitors and GLP1 Receptor Agonists: JACC Focus Seminar. J. Am. Coll. Cardiol..

[80] Davies M.J., D’Alessio D.A., Fradkin J., Kernan W.N., Mathieu C., Mingrone G., Rossing P., Tsapas A., Wexler D.J., Buse J.B. (2018). Management of Hyperglycemia in Type 2 Diabetes, 2018. A Consensus Report by the American Diabetes Association (ADA) and the European Association for the Study of Diabetes (EASD). Diabetes Care.

[81] Mashayekhi M., Nian H., Mayfield D., Devin J.K., Gamboa J.L., Yu C., Silver H.J., Niswender K., Luther J.M., Brown N.J. (2024). Weight Loss-Independent Effect of Liraglutide on Insulin Sensitivity in Individuals with Obesity and Prediabetes. Diabetes.

[82] Bardóczi A., Matics Z.Z., Turan C., Szabó B., Molnár Z., Hegyi P., Müller V., Horváth G. (2025). Efficacy of Incretin-Based Therapies in Obesity-Related Obstructive Sleep Apnea: A Systematic Review and Meta-Analysis of Randomized Controlled Trials. Sleep Med. Rev..

[83] Fortin S.M., Lipsky R.K., Lhamo R., Chen J., Kim E., Borner T., Schmidt H.D., Hayes M.R. (2020). GABA Neurons in the Nucleus Tractus Solitarius Express GLP-1 Receptors and Mediate Anorectic Effects of Liraglutide in Rats. Sci. Transl. Med..

[84] Kawatani M., Yamada Y., Kawatani M. (2018). Glucagon-like Peptide-1 (GLP-1) Action in the Mouse Area Postrema Neurons. Peptides.

[85] Imbernon M., Saponaro C., Helms H.C.C., Duquenne M., Fernandois D., Deligia E., Denis R.G.P., Chao D.H.M., Rasika S., Staels B. (2022). Tanycytes Control Hypothalamic Liraglutide Uptake and Its Anti-Obesity Actions. Cell Metab..

[86] Secher A., Jelsing J., Baquero A.F., Hecksher-Sørensen J., Cowley M.A., Dalbøge L.S., Hansen G., Grove K.L., Pyke C., Raun K. (2014). The Arcuate Nucleus Mediates GLP-1 Receptor Agonist Liraglutide-Dependent Weight Loss. J. Clin. Investig..

[87] Farr O.M., Sofopoulos M., Tsoukas M.A., Dincer F., Thakkar B., Sahin-Efe A., Filippaios A., Bowers J., Srnka A., Gavrieli A. (2016). GLP-1 Receptors Exist in the Parietal Cortex, Hypothalamus and Medulla of Human Brains and the GLP-1 Analogue Liraglutide Alters Brain Activity Related to Highly Desirable Food Cues in Individuals with Diabetes: A Crossover, Randomised, Placebo-Controlled Trial. Diabetologia.

[88] Salameh T.S., Rhea E.M., Talbot K., Banks W.A. (2020). Brain Uptake Pharmacokinetics of Incretin Receptor Agonists Showing Promise as Alzheimer’s and Parkinson’s Disease Therapeutics. Biochem. Pharmacol..

[89] Boer G.A., Hay D.L., Tups A. (2023). Obesity Pharmacotherapy: Incretin Action in the Central Nervous System. Trends Pharmacol. Sci..

[90] Moiz A., Filion K.B., Tsoukas M.A., Yu O.H., Peters T.M., Eisenberg M.J. (2025). Mechanisms of GLP-1 Receptor Agonist-Induced Weight Loss: A Review of Central and Peripheral Pathways in Appetite and Energy Regulation. Am. J. Med..

[91] van Bloemendaal L., Ten Kulve J.S., la Fleur S.E., Ijzerman R.G., Diamant M. (2014). Effects of Glucagon-like Peptide 1 on Appetite and Body Weight: Focus on the CNS. J. Endocrinol..

[92] Kadouh H., Chedid V., Halawi H., Burton D.D., Clark M.M., Khemani D., Vella A., Acosta A., Camilleri M. (2020). GLP-1 Analog Modulates Appetite, Taste Preference, Gut Hormones, and Regional Body Fat Stores in Adults with Obesity. J. Clin. Endocrinol. Metab..

[93] Dubé M.-C., D’Amours M., Weisnagel S.J. (2020). Effect of Liraglutide on Food Consumption, Appetite Sensations and Eating Behaviours in Overweight People with Type 1 Diabetes. Diabetes Obes. Metab..

[94] Martin C.K., Carmichael O.T., Carnell S., Considine R.V., Kareken D.A., Dydak U., Mattes R.D., Scott D., Shcherbinin S., Nishiyama H. (2025). Tirzepatide on Ingestive Behavior in Adults with Overweight or Obesity: A Randomized 6-Week Phase 1 Trial. Nat. Med..

[95] Heise T., DeVries J.H., Urva S., Li J., Pratt E.J., Thomas M.K., Mather K.J., Karanikas C.A., Dunn J., Haupt A. (2023). Tirzepatide Reduces Appetite, Energy Intake, and Fat Mass in People with Type 2 Diabetes. Diabetes Care.

[96] Nicolau J., Pujol A., Tofé S., Bonet A., Gil A. (2022). Short Term Effects of Semaglutide on Emotional Eating and Other Abnormal Eating Patterns among Subjects Living with Obesity. Physiol. Behav..

[97] Jacobsen L.V., Flint A., Olsen A.K., Ingwersen S.H. (2016). Liraglutide in Type 2 Diabetes Mellitus: Clinical Pharmacokinetics and Pharmacodynamics. Clin. Pharmacokinet..

[98] Blackman A., Foster G.D., Zammit G., Rosenberg R., Aronne L., Wadden T., Claudius B., Jensen C.B., Mignot E. (2016). Effect of Liraglutide 3.0  mg in Individuals with Obesity and Moderate or Severe Obstructive Sleep Apnea: The SCALE Sleep Apnea Randomized Clinical Trial. Int. J. Obes..

[99] Mifsud C.S., Kolla B.P., Rushlow D.R., Mansukhani M.P. (2025). The Impact of GLP-1 Agonists on Sleep Disorders: Spotlight on Sleep Apnea. Expert Opin. Pharmacother..

[100] Nauck M., Frid A., Hermansen K., Shah N.S., Tankova T., Mitha I.H., Zdravkovic M., Düring M., Matthews D.R., LEAD-2 Study Group (2009). Efficacy and Safety Comparison of Liraglutide, Glimepiride, and Placebo, All in Combination with Metformin, in Type 2 Diabetes: The LEAD (Liraglutide Effect and Action in Diabetes)-2 Study. Diabetes Care.

[101] Anderberg R.H., Richard J.E., Eerola K., López-Ferreras L., Banke E., Hansson C., Nissbrandt H., Berqquist F., Gribble F.M., Reimann F. (2017). Glucagon-Like Peptide 1 and Its Analogs Act in the Dorsal Raphe and Modulate Central Serotonin to Reduce Appetite and Body Weight. Diabetes.

[102] Kapitza C., Dahl K., Jacobsen J.B., Axelsen M.B., Flint A. (2017). Effects of Semaglutide on Beta Cell Function and Glycaemic Control in Participants with Type 2 Diabetes: A Randomised, Double-Blind, Placebo-Controlled Trial. Diabetologia.

[103] Gabery S., Salinas C.G., Paulsen S.J., Ahnfelt-Rønne J., Alanentalo T., Baquero A.F., Buckley S.T., Farkas E., Fekete C., Frederiksen K.S. (2020). Semaglutide Lowers Body Weight in Rodents via Distributed Neural Pathways. JCI Insight.

[104] Coskun T., Sloop K.W., Loghin C., Alsina-Fernandez J., Urva S., Bokvist K.B., Cui X., Briere D.A., Cabrera O., Roell W.C. (2018). LY3298176, a Novel Dual GIP and GLP-1 Receptor Agonist for the Treatment of Type 2 Diabetes Mellitus: From Discovery to Clinical Proof of Concept. Mol. Metab..

[105] Samms R.J., Coghlan M.P., Sloop K.W. (2020). How May GIP Enhance the Therapeutic Efficacy of GLP-1?. Trends Endocrinol. Metab..

[106] Brown E., Heerspink H.J.L., Cuthbertson D.J., Wilding J.P.H. (2021). SGLT2 Inhibitors and GLP-1 Receptor Agonists: Established and Emerging Indications. Lancet.

[107] Meier J.J. (2012). GLP-1 Receptor Agonists for Individualized Treatment of Type 2 Diabetes Mellitus. Nat. Rev. Endocrinol..

[108] Moiz A., Filion K.B., Tsoukas M.A., Yu O.H.Y., Peters T.M., Eisenberg M.J. (2025). The Expanding Role of GLP-1 Receptor Agonists: A Narrative Review of Current Evidence and Future Directions. eClinicalMedicine.

[109] Davies M.J., Bajaj H.S., Broholm C., Eliasen A., Garvey W.T., Roux C.W., le Lingvay I., Lyndgaard C.B., Rosenstock J., Pedersen S.D. (2025). Cagrilintide–Semaglutide in Adults with Overweight or Obesity and Type 2 Diabetes. N. Engl. J. Med..

[110] Kow C.S., Ramachandram D.S., Hasan S.S., Thiruchelvam K. (2025). Efficacy and Safety of GLP-1 Receptor Agonists in the Management of Obstructive Sleep Apnea in Individuals without Diabetes: A Systematic Review and Meta-Analysis of Randomized, Placebo-Controlled Trials. Sleep Med..

[111] Hirsch I.B., Parkin C.G., Cavaiola T.S., Bergenstal R.M. (2024). Use of Continuous Glucose Monitoring When Initiating Glucagon-like Peptide-1 Receptor Agonist Therapy in Insulin-Treated Diabetes. Diabetes Obes. Metab..

[112] Grunvald E., Shah R., Hernaez R., Chandar A.K., Pickett-Blakely O., Teigen L.M., Harindhanavudhi T., Sultan S., Singh S., Davitkov P. (2022). AGA Clinical Practice Guideline on Pharmacological Interventions for Adults with Obesity. Gastroenterology.

[113] Davies M.J., Aroda V.R., Collins B.S., Gabbay R.A., Green J., Maruthur N.M., Rosas S.E., Del Prato S., Mathieu C., Mingrone G. (2022). Management of Hyperglycemia in Type 2 Diabetes, 2022. A Consensus Report by the American Diabetes Association (ADA) and the European Association for the Study of Diabetes (EASD). Diabetes Care.

